# Evaluating the research capacity and culture amongst staff at a tertiary level teaching hospital in Rwanda

**DOI:** 10.1371/journal.pone.0314866

**Published:** 2024-12-05

**Authors:** Kara L. Neil, Daniella Rangira, Edouard Ngendahayo, Natalie McCall, Rafiki M. Gatera

**Affiliations:** 1 King Faisal Hospital Rwanda, Kigali, Rwanda; 2 Africa Health Sciences University, Kigali, Rwanda; 3 University of Toronto, Toronto, Ontario, Canada; 4 University of Global Health Equity, Butaro, Rwanda; World Health Organization, SOUTH SUDAN

## Abstract

**Background:**

Building research capacity can strengthen health systems through evidence-based interventions. However, evaluating the current research capacity and increasing it is a layered process that needs to consider different institutional structures, as well as internal factors. This study collects baseline data on the research capacity and culture at King Faisal Hospital Rwanda (KFH), a tertiary-level teaching hospital in Rwanda. It also proposes ways to further strengthen it and recommends ways for other institutions in Rwanda to strengthen research capacity.

**Methods:**

The Research Capacity and Culture Tool was distributed to full-time clinical and non-clinical KFH staff in September 2021. Participants were required to hold a position that minimally requires an Advanced Diploma. The quantitative survey data were analyzed in SPSS Version 27 and analyzed via descriptive statistics across all domains, including the individual, organizational, and team levels.

**Findings:**

152 participants completed the questionnaire. On a 5-point Likert scale, the highest ranked skills were designing questionnaires (3.34) and using digital referencing systems (3.29), while the lowest ranked skills were securing research funding (2.40) and writing for publication in peer-reviewed journals (2.46). Perceptions about the organizational level’s research system were overall stronger than those at the team level, with the weakest team-level system being having regular research forums and bulletins (2.14) and having digital tools for conducting research (2.14). Motivators to conducting research included skills development (87%) and career advancement (74%), while barriers included a lack of time (64%) and access to funding (56%).

**Discussion:**

To strengthen the research capacity and culture at KFH, focus should be on allocating tools, resources, and training opportunities to staff. Research should be integrated into staff job descriptions, with a time audit conducted to ensure they have adequate time for these activities. Finally, decentralizing research and ensuring team-level ownership will help with staff buy in.

## Introduction

Africa bears approximately 25% of the global burden of disease, yet it contributes to only 2% of global research output [1]. This disparity is attributed to several factors, including limited investment in research and development, with an average of only 0.4% of gross domestic products directed towards these efforts [2]. Additionally, various contexts across the continent face infrastructural deficiencies, a lack of comprehensive research policies, and significant gaps in research capacity, all of which impede scientific progress and innovation [1]. Despite these challenges, Africa holds immense potential in the field of data science and research, particularly given the pronounced equity gap that currently limits the representation of African populations in clinical datasets, which have the potential to lead to the development of contextually relevant clinical interventions [3].

Capacity building is essential to addressing research deficits across the continent, which are largely due to limited training opportunities, resources, and a shortage of mentorship models that could otherwise foster early-career researchers [4]. Improving local ownership of research activities and creating pathways for sustained skills development among researchers would support not only increased publication output and grant achievements but also facilitate the exploration of health issues that are locally relevant. African-led research initiatives may also enhance the cultural and policy resonance of study findings, making them more applicable and impactful for local populations, and enabling researchers to communicate findings within a context applicable to their patients and communities [5].

The benefits of capacity building, or “strengthening the ability of individuals, organizations, or systems to perform appropriate functions effectively, efficiently, and sustainably” are widespread and evidence-based, with research capacity building specifically having the potential to strengthen health systems through evidence-based findings and interventions [6]. However, evaluating the current research capacity and proposing interventions to increase it is a layered process that needs to consider different institutional structures (e.g., individual, team, institution), as well as internal factors (e.g., funding, research culture, partnerships, etc.) [6]. While research capacity building initiatives have typically been focused on individual training opportunities or time dedicated to research, recent studies argue for a more holistic approach that looks at the overall systems and infrastructure in place at various levels [6, 7]. There is also a need for further research on the linkage between research capacity building and its impact on patient outcomes in clinical settings within individual contexts.

In response to the need for research capacity evaluation tools that take a more holistic approach, Queensland Health and Griffith University (Australia) developed the Research Capacity and Culture (RCC) tool, which is an effective and validated tool for conducting research capacity needs assessments. The tool measures research capacity across the individual, team, and institution [8]. A study on the tool’s validity found that the RCC tool is a valid evaluation tool that can be used to identify research capacity building interventions and can also be adapted to different contexts [9]. The RCC tool has been utilized in various contexts beyond its initial development in Australia. For instance, a study conducted in the United Kingdom utilized a modified version of the RCC tool to assess allied health professionals’ perceptions of research capacity and culture within the National Health Service. This adaptation demonstrated the tool’s applicability in different healthcare settings and regions [10]. In the context of Rwanda, evaluating research capacity and proposing interventions in health institutions aligns with Rwanda’s national strategies. The Ministry of Health launched the “4x4 reform” to quadruple the health workforce in four years, with the aim to strengthen the quality and quantity of healthcare service delivery [11]. A significant part of this national strategy is to build the capacity of health science institutions to prepare the future workforce, which strongly emphasizes research capacity building, output, and translating research to evidence-based practice.

Located in Kigali, Rwanda, King Faisal Hospital Rwanda (KFH) is a multi-specialty, tertiary-level teaching hospital that provides a range of specialized healthcare services in Rwanda and the region. As a specialty referral and teaching hospital, it has a bed capacity of over 160 and employs over 600 staff members. KFH is establishing a degree-granting health sciences institution as part of the national 4x4 strategy and has invested significant resources into strengthening its research capacity and output as part of this transformation [12]. KFH has also invested significantly in research capacity building and output, as we demonstrated in its inaugural research day, with over 100 attendees and 47 abstracts [13]. As part of this development, this study collects baseline data on the existing research capacity and culture at KFH and proposes ways to further strengthen this capacity and culture. It also aims to provide recommendations for other health sciences institutions in Rwanda as they also seek to strengthen research capacity in alignment with the national strategy.

## Materials and methods

### Participants and sampling strategy

Participants included staff members on KFH payroll, or those on another organization’s payroll but seconded on a full-time basis to KFH as of September 2021. Participants meeting these criteria were also required to hold a position that minimally requires a minimum qualification of an Advanced Diploma (A1 degree) or the equivalent, as positions requiring these degree levels are typically expected to engage in research activities. This included both full-time clinical and non-clinical staff and consultant physicians. Among non-clinical staff, this included the hospital leadership and administration across all departments in positions requiring at least an A1 degree, including finance, procurement, education, quality assurance, and facilities. A total of 502 participants met this inclusion criteria and were sent the questionnaire.

### Data collection

Prior to collecting data, ethical approval was obtained from the KFH Institutional Review Board. The Research Capacity and Culture (RCC) Tool, developed by Queensland Health and Griffith University, was formatted as a Google survey, and circulated to participants via email (S1 File) [8]. Before completing the questionnaire, participants were required to give written consent to proceed. Participants were given one week to complete the survey over the first week of November 2021, and an email reminder was sent after five working days. The RCC tool phrasing was slightly modified to make questions more relevant to the national and organizational context (e.g., defining a team in the KFH context, highest education levels, and general phrasing), but the overall content of the questions remained the same.

### Data analysis

The quantitative survey data were analyzed in SPSS Version 27 (IBM Corp., USA). The descriptive statistics analysis, including means and median were performed across the Likert-scale statements (S2 File). The results at the organization and team levels were compared, and individual-level responses were also analyzed to extract common trends.

## Results

A total of 152 participants completed the questionnaire, with most participants holding a bachelor’s degree (38%) or master’s degree (30%). Furthermore, 10% held a medical degree and 24% held an advanced diploma. Participants indicated their current research activities, individual perceptions on their research capacity, organizational and team level support systems for research at KFH, as well as perceived barriers and motivators to conducting research. Table 1 outlines the demographics of participants.

**Table 1:** Participant professional profile.

Category	Response	n	%
# of Peer-Reviewed Publications	None	88	59.06%
	1–5	54	36.24%
	6–10	4	2.68%
	11–15	2	1.34%
	16–20	0	0.00%
	21 or Above	1	0.67%
Years of Professional Experience	1–5	34	22.37%
	6–10	46	30.26%
	11–15	45	29.61%
	16–20	15	9.87%
	21 or Above	12	7.89%

### Research activity

Participants were surveyed to ascertain to indicate whether research was part of their role expectations at KFH. 54% indicated research was part of their role expectations while 18% expressed uncertainty. Of those who indicated research as part of their role at KFH, the most cited provisions made for them by the hospital were dedicated time (45%), administrative support (42%), and training (35%). However, only 41% of the participants indicated having at least one peer-reviewed academic publication over the course of their careers. Furthermore, a majority (68%) indicated no research activity over the past 12 months, with 42% reporting no research activity throughout their career. Among those active in research, the primary activities they engaged in over the past 12 months included co-authoring a paper (18%), securing research funding (9%), and presenting research findings at a conference (9%).

### Individual perceptions on research capacity

Participants were asked to evaluate their perceived research capacity across different domains using a five-point Likert scale. Out of a maximum of five points, the highest ranked skills were linked to data collection and design, including collecting data (3.59), designing questionnaires (3.34), and using digital referencing systems (3.29). Meanwhile, the lowest ranked skills pertained to funding and finalizing research projects, including securing research funding (2.40), writing for publication in peer-reviewed journals (2.46), and mentoring less experienced researchers (2.78). Table 2 outlines the individual research skill level perceptions amongst participants.

**Table 2:** Individual research skill level perceptions.

Skill Area	Mean	Median	n	% Unsure
Collecting data (e.g. surveys, interviews)	3.59	4.00	146	6%
Designing questionnaires	3.34	3.50	144	8%
Using a computer referencing system (eg Endnote)	3.29	3.00	147	5%
Finding relevant literature	3.28	3.00	148	4%
Data interpretation and presentation	3.24	3.00	146	6%
Writing a research report	3.24	3.00	147	5%
Critically reviewing the literature	3.20	3.00	147	5%
Analysing quantitative research data	3.15	3.00	143	9%
Using computer data management systems	3.05	3.00	146	6%
Writing a research protocol	3.01	3.00	148	4%
Analysing qualitative research data	2.96	3.00	147	5%
Submitting an ethics application	2.94	3.00	145	7%
Providing advice to less experienced researchers	2.78	3.00	144	8%
Writing for publication in peer-reviewed journals	2.46	2.00	146	6%
Securing research funding	2.40	2.00	144	8%

### Organization & team level analysis

Participants assessed the available support systems at the organizational and team levels. The organizational level encompassed the entire hospital, while the team level represented the specific unit or department where the participant worked at the time of data collection. The findings largely align between the entire hospital and the individual teams. At the hospital level, the strongest areas include having a policy or plan for research development (3.44 versus 2.63 at the team level) and having senior managers that support research (3.26 versus 2.90 at the team level). Meanwhile, the strongest systems in place at the team level include having senior managers who support research (2.90) and ensuring that planning is guided by evidence (2.75). The weakest team-level systems included having software for conducting research (2.14) and having regular research forums and bulletins (2.14). Overall, respondents perceived the organizational level’s research systems to be stronger than those at the team level. Table 3 outlines the breakdown of organizational and team-level research systems in place.

**Table 3:** Organizational and team-level research systems.

	Organizational Level			Team Level			
Focus Area	Mean	N	% Unsure	Mean	N	% Unsure	P Value
Has a plan or policy for research development	3.44	141	11%	2.63	144	8%	0.000
Has senior managers that support research	3.26	137	15%	2.90	143	9%	0.017
Encourages research activities relevant to practice	3.13	138	14%	2.73	142	10%	0.007
Ensures planning is guided by evidence	2.93	132	20%	2.75	138	14%	0.137
Has funds, equipment or admin to support research activities	2.88	135	17%	2.26	136	16%	0.000
Supports a multi-disciplinary approach to research	2.82	140	12%	2.40	139	13%	0.007
Has adequate resources to support staff research training	2.77	140	12%	2.35	144	8%	0.004
Engages external partners (eg universities) in research	2.68	120	32%	2.32	130	22%	0.026
Has identified experts accessible for research advice	2.65	127	25%	2.40	134	18%	0.089
Has consumers (end users) involved in research	2.56	133	19%	2.53	141	11%	0.425
Accesses external funding for research	2.55	117	35%	2.15	119	33%	0.016
Has mechanisms to monitor research quality	2.53	120	32%	2.52	131	21%	0.469
Ensures staff career pathways are available in research	2.51	136	16%	2.68	147	5%	0.155
Supports the peer-reviewed publication of research	2.44	116	36%	2.24	127	25%	0.131
Supports applications for research scholarships/ degrees	2.43	131	21%	2.27	134	18%	0.188
Has software programs for analysing research data	2.23	116	36%	2.14	121	31%	0.312
Has regular forums/bulletins to present research findings	2.22	129	23%	2.14	130	22%	0.322

### Motivators and barriers to conducting research

Participants indicated motivators at KFH to conduct research across a multiple checkbox. The most frequently cited motivators included skills development (87%), career advancement (74%), and increased job satisfaction (60%). Meanwhile, the least cited motivators were having research as part of one’s job description (17%) and having colleagues who conduct research (20%). Table 4 outlines the breakdown of motivators to conducting research at KFH.

**Table 4:** Motivators to conduct research.

Barriers	Frequency (n = 151)	Percentage
To develop skills	131	87%
Career advancement	112	74%
Increased job satisfaction	91	60%
Problem identified that needs changing	80	53%
To keep the brain stimulated	66	44%
Grant funds	63	42%
Study or research scholarships available	59	39%
Mentors available to supervise	57	38%
Increased credibility	53	35%
Dedicated time for research	51	34%
Links to universities	51	34%
Desire to prove a theory	51	34%
Research encouraged by managers	43	28%
Opportunities to participate at own level	41	27%
Forms part of Postgraduate study	33	22%
Colleagues doing research	30	20%
Research written into role description	26	17%
Other: Not applicable (no research activity)	2	1%

In addition to identifying motivators, participants were also asked to indicate the barriers to conducting research at KFH. The most cited barrier included a lack of time (64%) and insufficient funding (56%) Similarly, more than half of respondents highlighted that other work responsibilities taking priority over research (52%). Table 5 outlines the frequency of barriers identified.

**Table 5:** Barriers to conducting research.

Barriers	Frequency (n = 151)	Percentage
Lack of time for research	96	64%
Lack of funds for research	84	56%
Other work roles take priority	78	52%
Lack of support from management	63	42%
Lack access to equipment for research	61	40%
Lack of administrative support	60	40%
Lack of software for research	60	40%
Lack of skills for research	53	35%
Lack of a coordinated approach to research	51	34%
Lack of staff to cover my other responsibilities	45	30%
Lack of library/internet access	34	23%
Other personal commitments	18	12%
Intimidated by research language	17	11%
Desire for work / life balance	16	11%
Intimidated by fear of getting it wrong	10	7%
Isolation	8	5%
Not interested in research	3	2%
Other: English language barriers	1	1%
Other: Not applicable (no research activity)	3	2%
Other: Lack of consideration of findings	1	1%

## Discussion

This study assessed baseline research capacity and culture among staff at KFH, revealing key insights aligned with broader trends in research engagement within similar contexts. The findings indicate that while many participants perceive research as an integral part of their role, a considerable gap remains between perceived role expectations and actual research engagement. Specifically, although over half the participants recognized research as part of their responsibilities, only 41% had a peer-reviewed publication, and 68% reported no research activity in the past year. High-rated skills among participants included designing questionnaires and using digital referencing tools, while securing funding and writing for publication were lower-rated areas. These findings are consistent with local studies in the region, which similarly highlight an interest in research amid barriers that limit effective engagement and output.

A central challenge identified in this study was the limited organizational support from an institutional perspective for research, particularly at the team level. In line with studies from other low- and middle-income countries (LMICs), barriers to research participation included time constraints, limited funding, and inadequate infrastructure. For example, a study in Tanzania found that, despite high interest, healthcare workers faced persistent barriers such as lack of funding and time, limited skills, and insufficient opportunities for research practice [14]. This Tanzanian study recommended a holistic approach to capacity building, including mentorship, policy support, and improved infrastructure—elements that also resonate with the findings at KFH. Addressing these barriers could enable healthcare professionals to actively engage in research that informs clinical practice, thereby advancing clinical care within healthcare settings [14–16].

Internationally, findings from high-income contexts highlight similar barriers but also suggest possible structural support systems that mitigate them. For instance, a study in Australia on research capacity in healthcare noted substantial differences in research activity across specialty teams, attributing these to the varying levels of funding and dedicated research time provided [17]. These studies suggest that specialty-specific research support structures could enhance engagement across diverse teams. In high-income settings, organizational frameworks are often in place to allocate resources, foster collaboration, and prioritize research activities. If these recommendations are adapted in settings like KFH, they could help address the identified gaps in team-level support and engagement.

From a clinical care perspective, previous studies have also noted that improved patient outcomes are correlated with research-active healthcare organizations [18–20]. Increased research activity has shown positive correlations with enhanced organizational efficiency, heightened staff satisfaction, diminished staff turnover rates, elevated patient satisfaction levels, and reduced mortality rates [18, 20, 21]. A study from the United Kingdom aimed to analyze the research of allied health professionals in a tertiary children’s hospital using the RCC tool, several obstacles were identified. These obstacles included time constraints, prioritization of clinical duties, and inadequate coverage for research tasks, while motivators for research engagement included skill enhancement, career progression, and heightened job fulfillment [18]. Furthermore, a previous systematic review examining whether there is an association between having a positive research culture in a health institution and better organizational performance, reported a positive association between research activity and organizational performance across all included studies [21]. This review provided evidence that a positive research culture and interventions directed at the health workforce are correlated with patient, staff, and organizational benefits [21].

Based on these findings, several recommendations can be made for strengthening research capacity at KFH and other similar institutions. First, implementing structured mentorship programs and targeted research training could support skill development in lower-rated skills areas, such as securing funding and writing for publication. Additionally, establishing dedicated time for research activities, alongside institutional policies that support research engagement, could bridge the gap between role expectations and actual research participation. Finally, fostering local and international collaborations can further support knowledge exchange and resource sharing, facilitating a research-conducive environment within LMICs. These initiatives could inform policy implementation efforts to strengthen research culture and productivity.

Some strengths and limitations arose in this study. A strength includes the study’s comprehensive approach, which aims to assess various aspects of research capacity and culture, including skills, perceptions, motivators, and barriers, providing a baseline understanding. This study also included both clinical and non-clinical staff, ensuring representation across different roles within the hospital, enhancing the characteristics of the sample. The use of the RCC tool, a standardized tool, is a strength since it ensures consistency in data collection and analysis, enhancing the reliability of the findings. Some limitations include the small sample size and low response rate, which may limit the generalizability of the findings, potential bias in self-report measures, and the cross-sectional nature of the study which implies that correlations do not indicate causation. Despite these limitations, the study offers valuable insights into improving research capacity and culture at KFH. Overall, this study’s findings align with existing literature on research capacity challenges in healthcare and underscore the critical role of organizational and policy-level interventions in fostering research engagement.

## Conclusions

Building upon the findings of this study and suggestions from previous literature, several recommendations can be proposed to strengthen research capacity at KFH and other health institutions in Rwanda. The findings of this study suggest that enhancing the research capacity and culture at KFH requires prioritizing the provision of essential tools, resources, and training for staff. Specifically, training programs that target areas identified as weaknesses in the study, such as writing publications and securing research funding, may help enhance research skills. This study also highlighted the importance of incorporating research responsibilities into staff job descriptions, accompanied by a time audit to guarantee sufficient time allocation. The hospital should consider exploring opportunities for external funding and partnerships to address barriers in research and allocating dedicated time for research activities. Furthermore, decentralizing research efforts and fostering team-level ownership are crucial for garnering staff support and engagement. In conclusion, by implementing these recommendations, KFH can further strengthen its research capacity and contribute to the advancement of healthcare knowledge and innovation in Rwanda. Insights from this study may ultimately lead to the fostering of a culture of research excellence and drive improvements in healthcare delivery and outcomes.

## Supporting information

S1 File Research capacity and culture survey tool. (DOCX)

S2 File Deidentified dataset. (XLSX)

## Abbreviations

KFH: King Faisal Hospital Rwanda
LMIC: Low- and middle-income country
RCC: Research Capacity and Culture
